# Supplementary material for: Tau uptake by human neurons depends on receptor LRP1 and kinase LRRK2
Source: EMBO J. 2025 Aug 11;44(18):5149–86. doi: 10.1038/s44318-025-00514-0 (PMC12436637; doi:10.1038/s44318-025-00514-0)
Supplement: Supplementary file 8 — Expanded View Figures [file 44318_2025_514_MOESM8_ESM.pdf]

Expanded View Figures

Figure EV1. Development and optimisation of CRISPR screens for tau uptake in human iPSC-derived excitatory neurons.

(**A**) Construct of KOLF2-C1 Cas9 cell line. KOLF2-C1 parent cell line genetically engineered at the AAVS1 locus to constitutively express humanised Cas9 with N-terminal FLAG tag (three repeats) and SV40 nuclear localisation signal (NLS) and C-terminal nucleoplasmin NLS from CAG promoter (CMV/chicken β-actin promoter). (**B**) Gene expression analysis of iPSC-derived neuronal progenitor cells from KOLF2-C1 (parental line) and KOLF2-C1 Cas9 (constitutively expressing Cas9), 35 days after induction. Selected genes whose expression is specific or enriched in particular regions and/or cell types are shown from a panel of 200 genes (see Experimental Procedures for details). (**C**) Analysis of the proportion of MAP2-positive KOLF2-C1 Cas9 neurons 60 days after induction. Neurons were dissociated into single cells and immunostained using MAP2 antibody conjugated to PE and analysed by flow cytometry. To establish threshold levels for MAP2-positive neurons (bold horizontal bar), unstained control neurons were analysed. (**D**, **E**) Optimising the concentration of tau protein to achieve saturating levels of extracellular tau uptake during acute treatment, measured by flow cytometry. Graph reports percentage of tau-positive neurons over time after tau incubation initiation, with concentration of tau protein as indicated. Data are shown from one representative experiment. (**F**) Percentage of whole-genome CRISPR gRNA library lentivirus-transduced (BFP+) neurons in tau uptake FACS screens. A monomeric tau uptake screen was carried out in duplicate, and a fibrillar tau uptake screen in triplicate. (**G**) Total number of lentivirus-transduced (BFP+) neurons collected by flow cytometry in each screen (left axis) and corresponding library coverage (n. cells/n. gRNAs) (right axis). (**H**) Percentage of lentivirus-transduced neurons collected from monomeric and fibrillar tau uptake screens that are transferrin positive and tau negative. (**I**) Total number of transferrin-positive, tau-negative lentivirus-transduced neurons collected by flow cytometry in each screen (left axis) and corresponding library coverage (n. cells/n. gRNAs) (right axis). (**J**) Distribution of median normalised log2 guide RNA counts from transferrin positive and either tau positive (+) or negative (−) neurons collected from monomeric (purple; two screens) and fibrillar (orange; three screens) tau uptake screens. (**K**) Percentage of CRISPR gRNA detected in transferrin-positive and either tau-positive (POSPOS) or negative (NEGPOS) neurons collected from tau uptake FACS screens.

 © The Author(s)

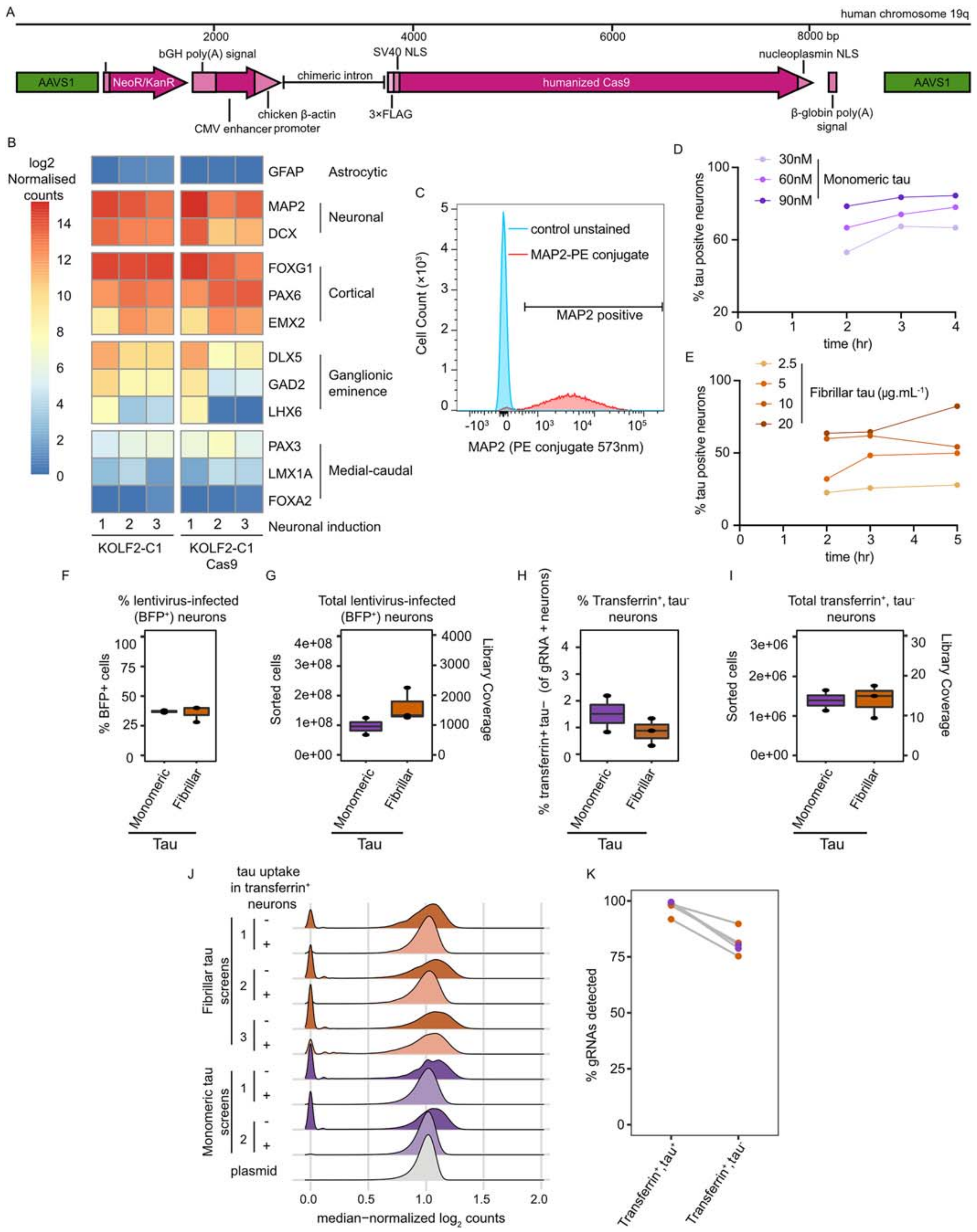
A
human chromosome 19q
2000
4000
6000
8000 bp
bGH poly(A) signal
SV40 NLS
nucleoplasmin NLS
chimeric intron
AAVS1
NeoR/KanR
humanized Cas9
AAVS1
chicken β-actin
CMV enhancer promoter
3×FLAG
β-globin poly(A) signal
B
log2 Normalised counts
GFAP
Astrocytic
MAP2
DCX
Neuronal
FOXG1
PAX6
EMX2
Cortical
DLX5
GAD2
LHX6
Ganglionic eminence
PAX3
LMX1A
FOXA2
Medial-caudal
Neuronal induction
KOLF2-C1
KOLF2-C1 Cas9
C
control unstained
MAP2-PE conjugate
MAP2 positive
Cell Count (×10³)
MAP2 (PE conjugate 573nm)
D
30nM
60nM
90nM
Monomeric tau
% tau positive neurons
time (hr)
E
2.5
5
10
20
Fibrillar tau (µg.mL⁻¹)
% tau positive neurons
time (hr)
F
% lentivirus-infected (BFP⁺) neurons
% BFP+ cells
Monomeric
Fibrillar
Tau
G
Total lentivirus-infected (BFP⁺) neurons
Sorted cells
Library Coverage
Monomeric
Fibrillar
Tau
H
% Transferrin⁺, tau⁻ neurons
% transferrin+ tau− (of gRNA + neurons)
Monomeric
Fibrillar
Tau
I
Total transferrin⁺, tau⁻ neurons
Sorted cells
Library Coverage
Monomeric
Fibrillar
Tau
J
tau uptake in transferrin⁺ neurons
Fibrillar tau screens
Monomeric tau screens
plasmid
median−normalized log₂ counts
K
% gRNAs detected
Transferrin⁺, tau⁺
Transferrin⁺, tau⁻

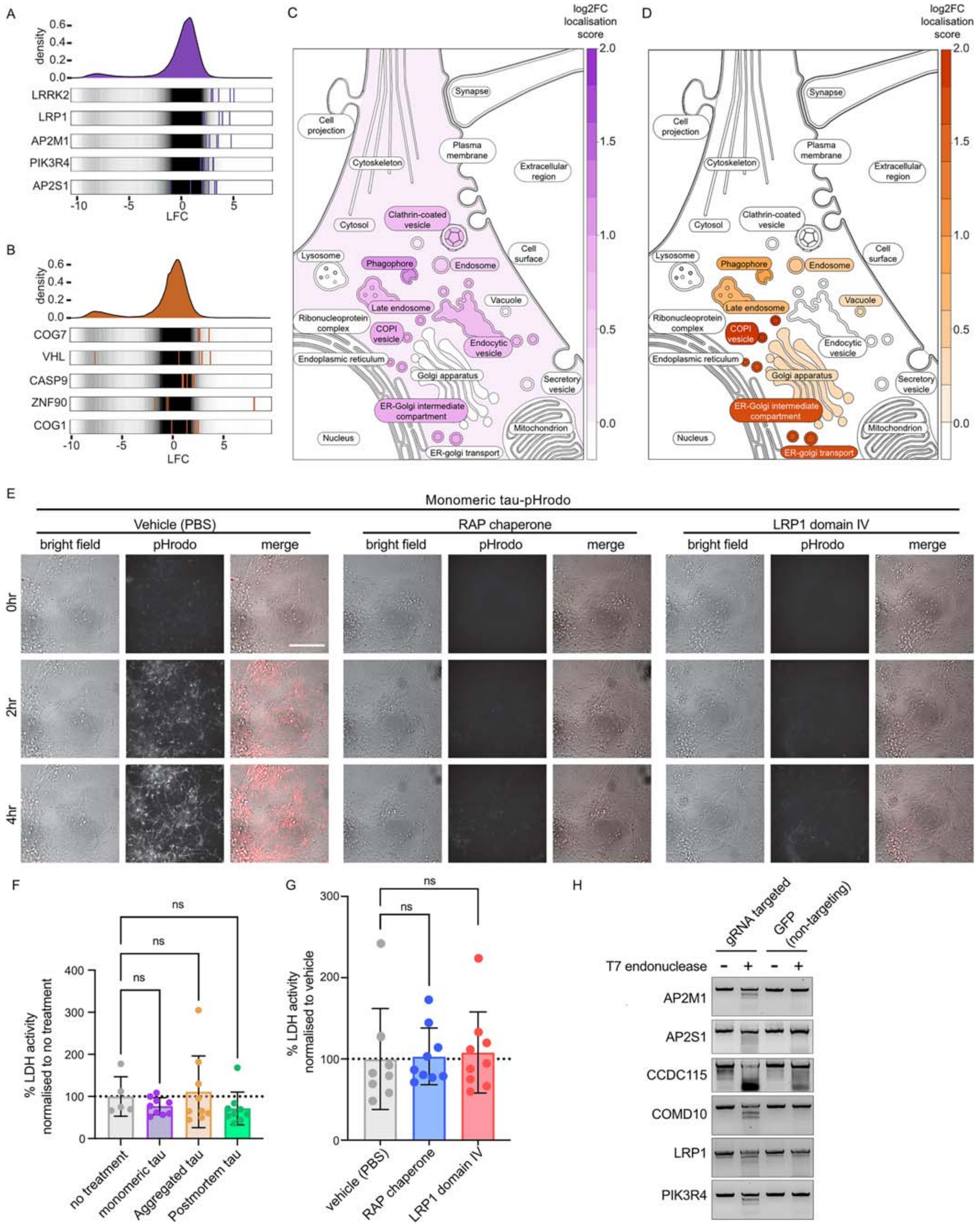
A
density
LRRK2
LRP1
AP2M1
PIK3R4
AP2S1
LFC
B
COG7
VHL
CASP9
ZNF90
COG1
C
log2FC localisation score
Synapse
Cell projection
Plasma membrane
Cytoskeleton
Extracellular region
Clathrin-coated vesicle
Cytosol
Cell surface
Lysosome
Phagophore
Endosome
Late endosome
Vacuole
Ribonucleoprotein complex
COPI vesicle
Endocytic vesicle
Endoplasmic reticulum
Golgi apparatus
Secretory vesicle
ER-Golgi intermediate compartment
Mitochondrion
Nucleus
ER-golgi transport
D
E
Monomeric tau-pHrodo
Vehicle (PBS)
RAP chaperone
LRP1 domain IV
bright field
pHrodo
merge
0hr
2hr
4hr
F
% LDH activity normalised to no treatment
ns
no treatment
monomeric tau
Aggregated tau
Postmortem tau
G
% LDH activity normalised to vehicle
vehicle (PBS)
RAP chaperone
LRP1 domain IV
H
gRNA targeted
GFP (non-targeting)
T7 endonuclease
AP2M1
AP2S1
CCDC115
COMD10
LRP1
PIK3R4

© The Author(s)

Figure EV2. Identification of genes involved in the uptake of structurally distinct forms of tau by human cortical neurons via low-pH intracellular compartments.

(**A**, **B**) CRISPR gRNA log fold change (LFC) between transferrin positive and either tau positive (+) or negative (−) neurons collected from monomeric (**A**) and fibrillar (**B**) tau uptake screens analysed using the MAGeCK algorithm. Guides for the five highest-ranked genes (gene names indicated) are highlighted on the guide density plots. Genes required for uptake of monomeric (**C**), fibrillar (**D**) tau code for proteins with a significantly higher than random localisation score in particular cellular compartments in the COMPARTMENTS dataset (FDR <0.05). Significantly enriched compartments are coloured based on the strength of enrichment (log2 fold change), whereas non-significant compartments are left white. (**E**) Time-lapse (0- to 4-h) images showing uptake into iPSC-derived human neurons (60 days after induction) of extracellular monomeric tau conjugated to a pH-sensitive dye (inverse relationship between fluorescence and pH). Neurons and tau protein were individually pre-incubated with either 10 nM RAP chaperone, 100 nM LRP1 domain IV peptide or vehicle control (PBS) for 3 h prior to combining the tau incubations with neurons and live imaging of neuronal uptake of tau. Bright-field (grey scale in merge) and pH-sensitive fluorescent signal (pHrodo; red in merge) were captured using automated imaging on the Opera-Phenix platform (Perkin Elmer). Scale bar, 100 µm. (**F**) None of the forms of tau were acutely toxic to neurons over a 16 hr period, as measured by extracellular LDH activity (three wells per treatment), in the presence of 25 nM Monomeric tau, 150 nM (monomer molar equivalent) fibrillar or post-mortem tau. (**G**) Extracellular LDH activity was also used to determine neuronal viability in the presence of vehicle (PBS), 10 nM RAP chaperone or 100 nM LRP1 domain IV peptide (after treatment for one week; >8 wells per treatment, across two biological replicates). Error bars indicate SD. Significance was determined using one-way ANOVA (**p* < 0.05, ***p* < 0.01, Dunnett's test for multiple comparisons). (**H**)The T7 endonuclease assay was used to confirm CRISPR guide RNA targeting. Assays were performed on amplified genomic DNA regions containing the target site for CRISPR gRNAs to genes indicated in the presence of targeting gRNA and the non-targeting GFP control.

© The Author(s)

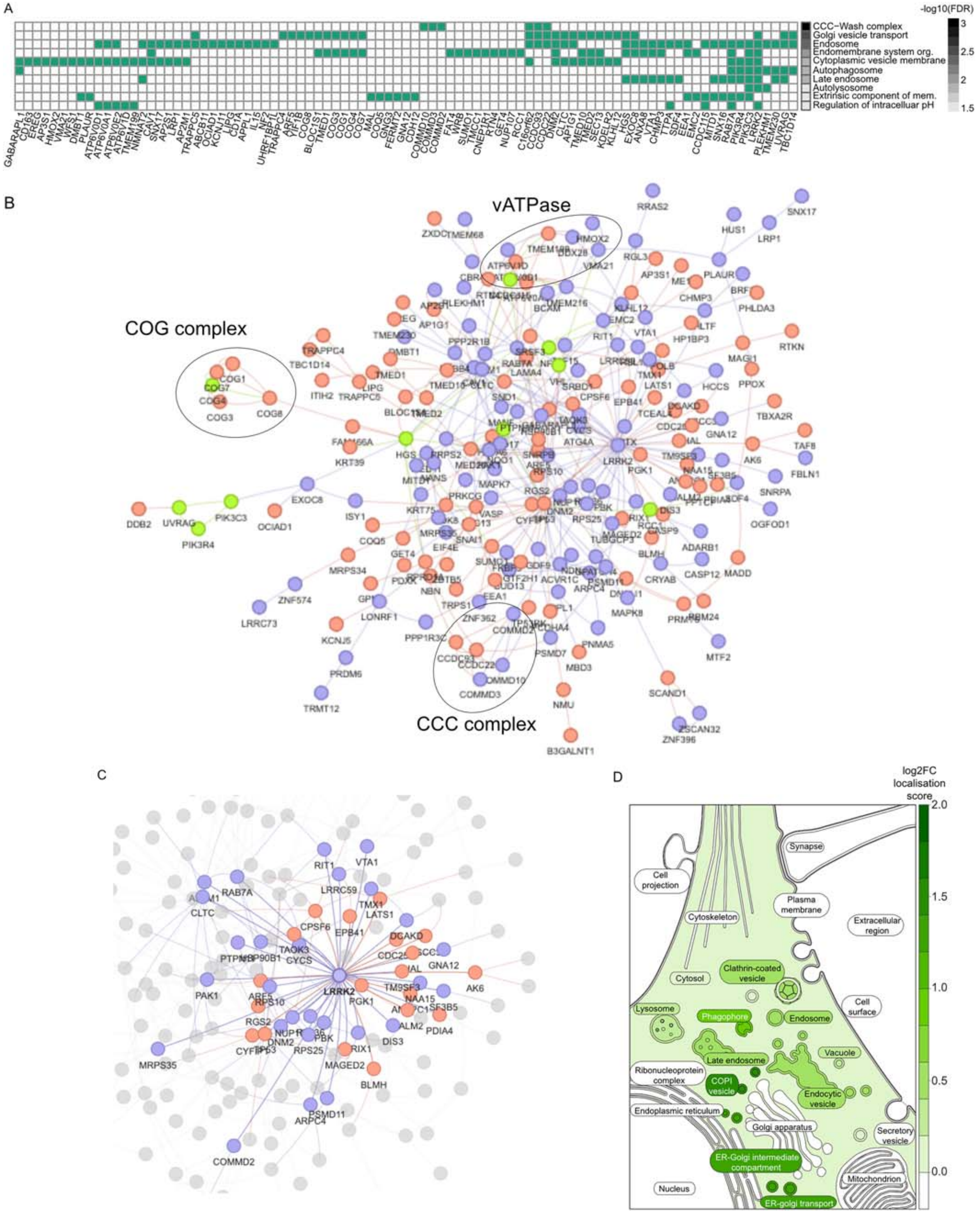
A
-log10(FDR)
CCC-Wash complex
Golgi vesicle transport
Endosome
Endomembrane system org.
Cytoplasmic vesicle membrane
Autophagosome
Late endosome
Autolysosome
Extrinsic component of mem.
Regulation of intracelluar pH
3
2.5
2
1.5
B
vATPase
COG complex
CCC complex
LRRK2
C
LRRK2
D
log2FC localisation score
2.0
1.5
1.0
0.5
0.0
Synapse
Cell projection
Plasma membrane
Cytoskeleton
Extracellular region
Cytosol
Clathrin-coated vesicle
Cell surface
Lysosome
Phagophore
Endosome
Late endosome
Vacuole
Ribonucleoprotein complex
COPI vesicle
Endocytic vesicle
Endoplasmic reticulum
Golgi apparatus
Secretory vesicle
ER-Golgi intermediate compartment
Mitochondrion
Nucleus
ER-golgi transport

 © The Author(s)

◀ **Figure EV3. Comparison of genes required for monomeric and fibrillar tau entry by human cortical neurons.**

(**A**) Heat map showing a representative selection of significantly enriched terms annotating the 431 genes significantly enriched for either monomeric or fibrillar tau uptake. The black colour scale indicates the significance level (−log10 FDR). Rows are sorted in order of significance, also see Dataset EV3. The central heatmap shows which of the enriched genes is annotated with each term. (**B**) PSICQUIC-derived network of experimentally validated physical interactions between proteins encoded by genes identified in either screen. Nodes are colour-coded depending on whether the corresponding gene was identified as required for monomeric (purple) or fibrillar (orange) tau uptake, or both (green). Some notable complexes are highlighted with circles. Interactions disconnected from the main network are not included. (**C**) The direct interactors of LRRK2, the most connected node, are highlighted in the protein interaction network; indirect interactions and genes unconnected to LRRK2 appear in grey. (**D**) Genes required for uptake of either monomeric or fibrillar tau code for proteins with a significantly higher than random localisation score in particular cellular compartments in the COMPARTMENTS dataset (FDR <0.05). Significantly enriched compartments are coloured based on the strength of enrichment (log2 fold change), whereas non-significant compartments are left white.

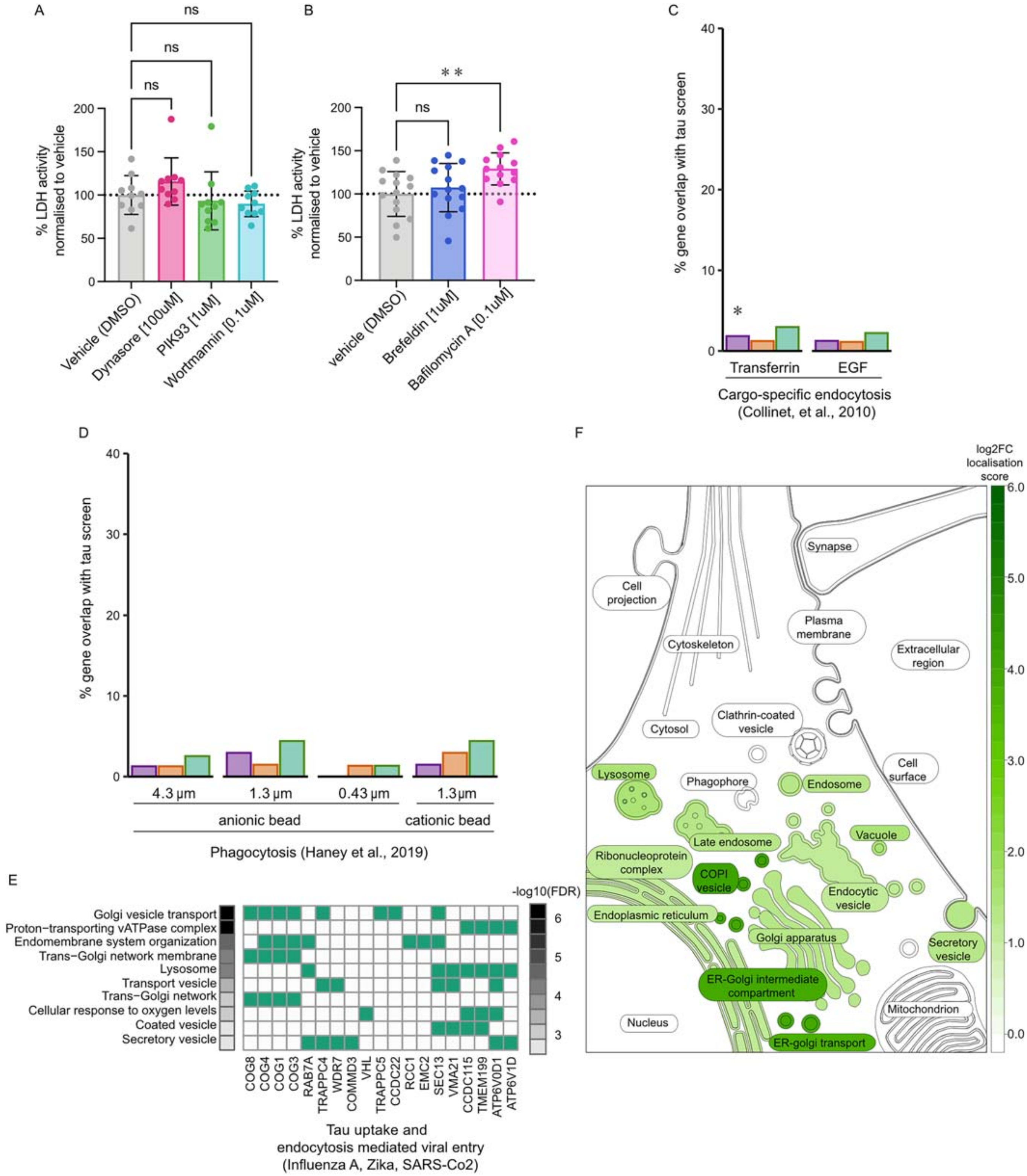
A
% LDH activity normalised to vehicle
ns
ns
ns
Vehicle (DMSO)
Dynasore [100uM]
PIK93 [1uM]
Wortmannin [0.1uM]
B
% LDH activity normalised to vehicle
ns
**
vehicle (DMSO)
Brefeldin [1uM]
Bafilomycin A [0.1uM]
C
% gene overlap with tau screen
*
Transferrin
EGF
Cargo-specific endocytosis (Collinet, et al., 2010)
D
% gene overlap with tau screen
4.3 µm
1.3 µm
0.43 µm
1.3µm
anionic bead
cationic bead
Phagocytosis (Haney et al., 2019)
E
-log10(FDR)
Golgi vesicle transport
Proton−transporting vATPase complex
Endomembrane system organization
Trans−Golgi network membrane
Lysosome
Transport vesicle
Trans−Golgi network
Cellular response to oxygen levels
Coated vesicle
Secretory vesicle
COG8
COG4
COG1
COG3
RAB7A
TRAPPC4
WDR7
COMMD3
VHL
TRAPPC5
CCDC22
RCC1
EMC2
SEC13
VMA21
CCDC115
TMEM199
ATP6V0D1
ATP6V1D
Tau uptake and endocytosis mediated viral entry (Influenza A, Zika, SARS-Co2)
F
log2FC localisation score
Synapse
Cell projection
Plasma membrane
Cytoskeleton
Extracellular region
Clathrin-coated vesicle
Cytosol
Cell surface
Lysosome
Phagophore
Endosome
Late endosome
Vacuole
Ribonucleoprotein complex
COPI vesicle
Endocytic vesicle
Endoplasmic reticulum
Golgi apparatus
Secretory vesicle
ER-Golgi intermediate compartment
Mitochondrion
Nucleus
ER-golgi transport

 © The Author(s)

Figure EV4. Cellular mechanisms for tau uptake and processing by human excitatory neurons in relation to endocytosis and viral entry, CRISPR screens.

(**A**) Extracellular LDH activity was used to determine neuronal viability in the presence of vehicle (0.1% [v/v] DMSO), 100 µM Dynasore, 1 µM PIK93 or 0.1 µM Wortmannin (after treatment for one week) (**B**), and for 1 µM Brefeldin or 0.1 µM Bafilomycin A (after treatment for 24 hr) (I). Only Bafilomycin A had a modest effect on neuronal viability (ten wells per treatment). Error bars indicate SD. Significance was determined using one-way ANOVA (*$p < 0.05$, **$p < 0.01$, Dunnett's test for multiple comparisons). (**C**) Neuronal uptake of tau protein requires cargo-specific endocytic adaptors. Comparison of sets of genes required for tau uptake with genes identified in screens for endocytosis of either transferrin or epidermal growth factor (EGF), shows significant overlap between monomeric tau uptake and transferrin endocytosis, but no other significant overlaps between the gene sets. (**D**) Genes involved in tau uptake do not significantly overlap with sets of genes identified as phagocytosis regulators in screens performed with distinct substrates, varying in their diameter (µm) and charge. (**E**) Heatmap showing functional annotations enriched among genes required for the uptake of either form of tau and Influenza A, Zika or SARS-CoV-2 virus entry (representative term selection). (**F**) Genes required for either monomeric or fibrillar tau uptake and Influenza A, Zika or SARS-CoV-2 viral entry code for proteins with a significantly higher than random localisation score in particular cellular compartments in the COMPARTMENTS dataset (FDR <0.05). Significantly enriched compartments are coloured based on the strength of enrichment (log2 fold change), whereas non-significant compartments are left white.

© The Author(s)

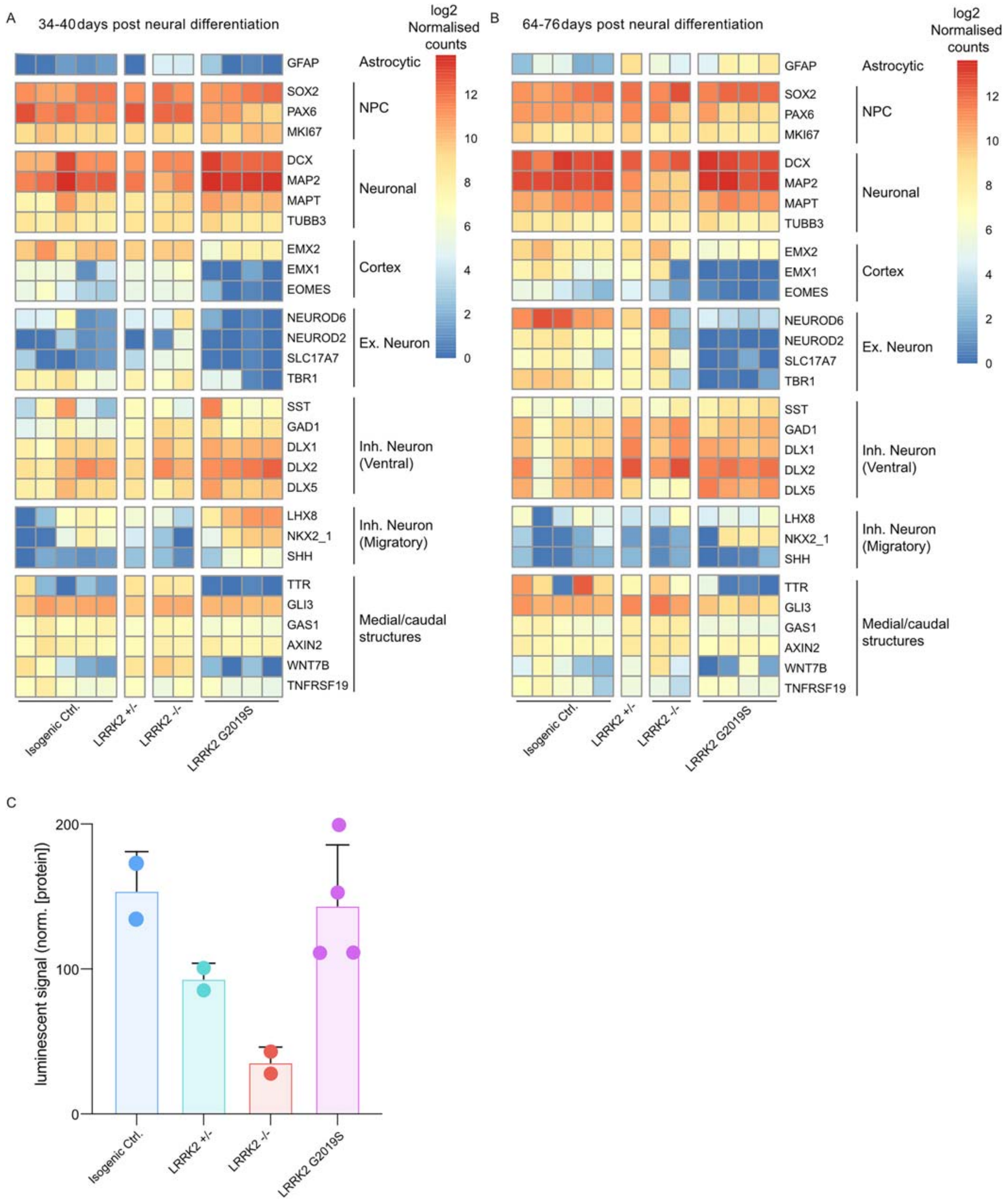
A
34-40 days post neural differentiation
log2 Normalised counts
GFAP
SOX2
PAX6
MKI67
DCX
MAP2
MAPT
TUBB3
EMX2
EMX1
EOMES
NEUROD6
NEUROD2
SLC17A7
TBR1
SST
GAD1
DLX1
DLX2
DLX5
LHX8
NKX2_1
SHH
TTR
GLI3
GAS1
AXIN2
WNT7B
TNFRSF19
Astrocytic
NPC
Neuronal
Cortex
Ex. Neuron
Inh. Neuron (Ventral)
Inh. Neuron (Migratory)
Medial/caudal structures
Isogenic Ctrl.
LRRK2 +/-
LRRK2 -/-
LRRK2 G2019S
B
64-76 days post neural differentiation
C
luminescent signal (norm. [protein])
0
100
200

 © The Author(s)

Figure EV5. Neuronal gene expression and LRRK2 protein levels in iPSC-derived cortical neurons with LRRK2 gain and loss of function mutations.

(**A**, **B**) Gene expression (Nanostring) analysis of iPSC-derived neuronal progenitor cells from KOLF2-C1 (parental line; Isogenic control), LRRK2 heterozygous null (LRRK2 −/+), LRRK2 homozygous null (LRRK2 −/−) and LRRK2 heterozygous G2019S, 35 (**A**) and between 64–76 (**B**) days after induction. Selected genes whose expression is specific or enriched in particular regions and/or cell types are shown from a panel of 200 genes (see Experimental Procedures for details), demonstrating that the inductions generated primarily cortical progenitor cells, with some ventral interneuron progenitor cells, which subsequently generate excitatory and inhibitory neurons. (**C**) LRRK2 protein levels in iPSC from KOLF2-C1 (parental line; Isogenic control), LRRK2 heterozygous null (LRRK2+/−), LRRK2 homozygous null (LRRK2−/−) and LRRK2 heterozygous G2019S, measured by AlphaLISA assay (see Methods for details; at least two wells per genotype). LRRK2 +/− cells have reduced LRRK2 protein relative to the parental cell, whereas LRRK2 −/− cells have protein around the detection threshold of the assay.

© The Author(s)

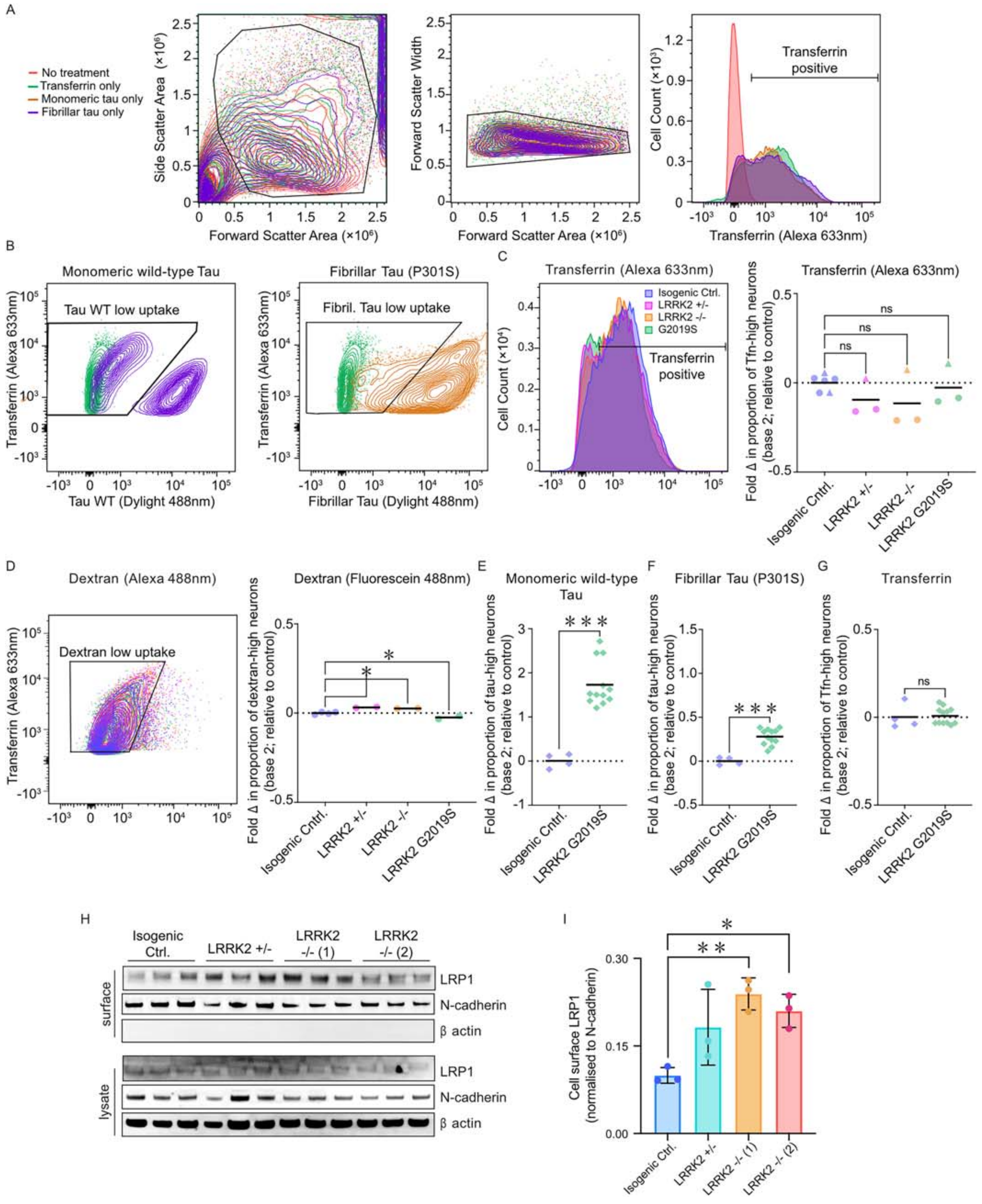
A
No treatment
Transferrin only
Monomeric tau only
Fibrillar tau only
Side Scatter Area (×10⁶)
Forward Scatter Area (×10⁶)
Forward Scatter Width
Cell Count (×10³)
Transferrin positive
Transferrin (Alexa 633nm)
B
Monomeric wild-type Tau
Tau WT low uptake
Transferrin (Alexa 633nm)
Tau WT (Dylight 488nm)
Fibrillar Tau (P301S)
Fibril. Tau low uptake
Fibrillar Tau (Dylight 488nm)
C
Transferrin (Alexa 633nm)
Isogenic Ctrl.
LRRK2 +/-
LRRK2 -/-
G2019S
Cell Count (×10⁴)
Transferrin positive
Fold Δ in proportion of Tfn-high neurons (base 2; relative to control)
ns
Isogenic Cntrl.
LRRK2 +/-
LRRK2 -/-
LRRK2 G2019S
D
Dextran (Alexa 488nm)
Dextran low uptake
Dextran (Fluorescein 488nm)
Fold Δ in proportion of dextran-high neurons (base 2; relative to control)
E
Monomeric wild-type Tau
Fold Δ in proportion of tau-high neurons (base 2; relative to control)
***
F
Fibrillar Tau (P301S)
G
Transferrin
H
Isogenic Ctrl.
LRRK2 +/-
LRRK2 -/- (1)
LRRK2 -/- (2)
surface
lysate
LRP1
N-cadherin
β actin
I
Cell surface LRP1 (normalised to N-cadherin)
LRRK2 -/- (1)
LRRK2 -/- (2)

 © The Author(s)

Figure EV6. Uptake of transferrin and dextran by LRRK2 gain and loss of function mutation-expressing neurons.

(**A**) Flow cytometry gating strategy for detection of transferrin and indicated cargo protein uptake into iPSC-derived isogenic control neurons (65 days after induction). Left panels show discrimination based on scatter parameters of neurons incubated with either no treatment (red) or extracellular transferrin (green), monomeric tau (orange) or fibrillar tau (blue). Black polygons and bold horizontal bars indicate gates applied to isolate the population of single neurons that endocytose transferrin. To establish threshold levels for positive protein uptake (bold horizontal bars), neurons without transferrin (red) or tau (green contours) incubation were analysed.
(**B**) Fluorescent intensity of either monomeric or fibrillar Dylight 488 nm low tau protein uptake populations are indicated by black polygons. (**C**) Isogenic control, LRRK2 heterozygous (+/−), homozygous (−/−) null or LRRK2 G2019S mutant neurons (65 days after induction) were incubated with 90 nM transferrin (Tfn) Alexa 633 nm or Dextran Fluorescein 488 nm (**D**) for 4 h before dissociation into single cells and analysis by flow cytometry. For each genotype and cell line indicated, the percentage of cells with low protein uptake (indicated by the black outlined polygons) was compared to the mean (dashed line) of protein uptake of control neurons and displayed as fold change (log2). Significance was determined using one-way ANOVA (*$p < 0.05$, **$p < 0.01$, Dunnett's test for multiple comparisons; circles and triangles represent independent experiments; $n = >3$). (**E–G**) Biological replicate flow cytometry assays of the effect of LRRK2 G2019S mutation on neuronal uptake of extracellular tau. Isogenic control and LRRK2 G2019S mutant neurons (65 days after induction) were incubated with 90 nM transferrin (Tfn) Alexa 633 nm and either monomeric wild-type (**E**), or fibrillar P301S (**F**) tau Dylight 488 nm, or transferrin alone (**G**), for 4 h before dissociation into single cells and analysis by flow cytometry (Thermo Fisher Attune CytPix). For LRRK2 G2019S mutant neurons, the percentage of cells with low protein uptake (in neurons gated for transferrin uptake) was compared to the mean (dashed line) of protein uptake of control neurons and displayed as fold change (log2). Significance was determined using an unpaired *t*-test (***$p < 0.001$; $n = 4$ for controls and $n = 12$ for LRRK2 G2019S; neurons generated from two and four independent neural inductions from control and LRRK2 G2019S iPSCs, respectively). (**H**) LRRK2 homozygous null neurons have increased levels of tau receptor protein LRP1 at the neuronal surface compared with isogenic controls. LRRK2 heterozygous and homozygous null neurons (60 days after induction) were surface biotinylated, and Neutravidin-coated particles were used to capture biotinylated membrane proteins. Surface abundance of indicated proteins were measured by immunoblotting. (**I**) Cell surface levels of LRP1 normalised to N-cadherin are shown for each genotype. Significance was determined using one-way ANOVA (*$p < 0.05$, **$p < 0.01$, Dunnett's test for multiple comparisons, $n = 3$).

© The Author(s)

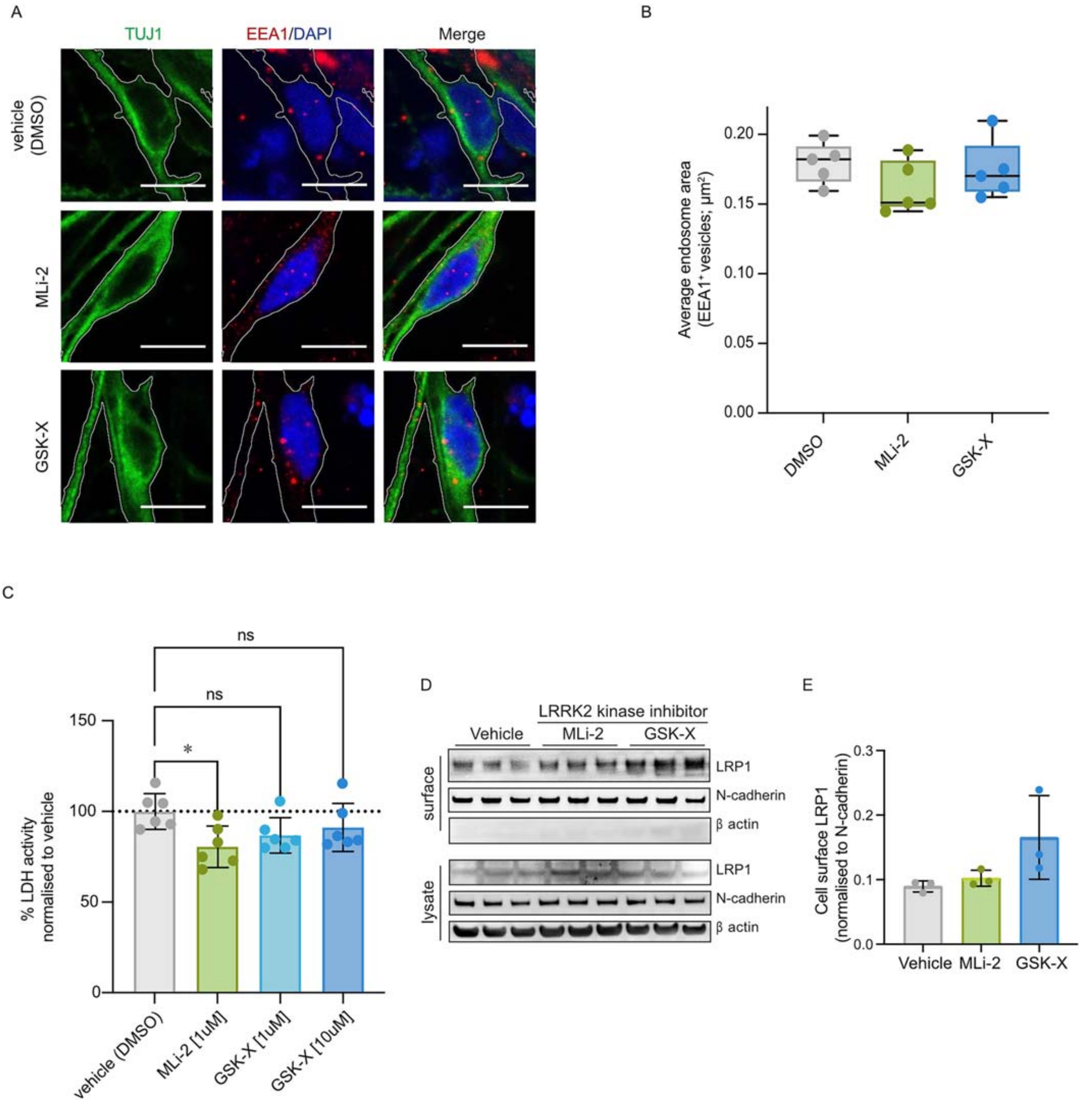

Figure EV7. Effect of LRRK2 kinase activity inhibitors on the endolysosomal system of human cortical neurons.

(**A**, **B**) Neurons treated with LRRK2 kinase activity inhibitors do not exhibit an endosomal or lysosomal phenotype. (**A**) Representative immunohistochemistry of neurons (60 days after induction) treated for 1 week with vehicle control (0.1% [v/v] DMSO), 1 µM MLi-2 or 10 µM GSK-X, immunostained for neuronal β3-tubulin (TUJ1, green), early endosomes (EEA1; red) and nuclei were counterstained (DAPI, blue). Scale bar, 10 µm. (**B**) No significant changes in the average size of early endosomes (EEA positive vesicles; µm^2) in neurons compared with isogenic control (n = >5 images). (**C**) Extracellular LDH activity was used to assess neuronal viability in the presence of vehicles (0.1% [v/v] DMSO), 1 µM MLi-2, 1 µM or 10 µM GSK-X (GSK3357679A; treatment for 1 week). Only 1 µM MLi-2 had a modest effect on neuronal viability (six wells per treatment). Error bars indicate SD. Significance was determined using one-way ANOVA (*$p < 0.05$, Dunnett's test for multiple comparisons). (**D**) Effect of inhibition of LRRK2 kinase activity on neuronal surface levels of tau receptor protein LRP1. Neurons were pre-incubated with either 1 µM MLi-2, 10 µM GSK-X or vehicle control (0.1% [v/v] DMSO) for one week prior to cell surface biotinylation (61 days after induction), followed by capture of biotinylated membrane proteins using Neutravidin-coated particles. Surface abundance of indicated proteins were examined by immunoblotting. (**E**) Cell surface levels of LRP1 normalised to N-cadherin are shown for each of the treatments (three replicate treatments).

 © The Author(s)
